# Nutritional support and prevention of post-intensive care syndrome: the Italian SIAARTI survey

**DOI:** 10.1186/s44158-023-00132-4

**Published:** 2023-11-07

**Authors:** Antonella Cotoia, Michele Umbrello, Fiorenza Ferrari, Vincenzo Pota, Francesco Alessandri, Andrea Cortegiani, Silvia De Rosa

**Affiliations:** 1 Department of Medical and Surgical Sciences, University of Foggia, Foggia, Italy; 2 Anesthesia and Intensive Care Unit, Policlinico Riuniti Foggia, Foggia, Italy; 3 Department of Intensive Care and Anaesthesia, ASST Ovest Milanese, Legnano Hospital, Milan, Italy; 4 Department of Anesthesia, Critical Care and Emergency, Fondazione IRCCS Ca’ Granda, Ospedale Maggiore Policlinico, Milan, Italy; 5 International Renal Research Institute of Vicenza (IRRIV), Ospedale San Bortolo, Vicenza, Italy; 6 Department of Woman, Child, General, and Specialty Surgery, L. Vanvitelli University of Campania, Naples, Italy; 7 Department of General and Specialistic Surgery, ‘Sapienza’ University of Rome, Intensive Care Unit Policlinico Umberto I Hospital, Rome, Italy; 8 Department of Surgical, Oncological and Oral Science (Di.Chir.On.S), University of Palermo, Palermo, Italy; 9 Department of Anaesthesia, Intensive Care and Emergency, Policlinico Paolo Giaccone, Palermo, Italy; 10 Centre for Medical Sciences-CISMed, University of Trento, Trento, Italy; 11 Anesthesia and Intensive Care, Santa Chiara Regional Hospital, APSS, Trento, Italy

**Keywords:** Critical care, Critical illness, Nutrition, Nutrition assessment, Nutrition management, SIAARTI

## Abstract

**Background:**

Malnutrition and muscle wasting are common in ICU patients and predict adverse patient-centered outcomes. The Italian Society of Anesthesia Analgesia Resuscitation and Intensive Care (SIAARTI) conducted a nationwide survey to identify the nutritional practices in the Italian ICUs and to plan future, training interventions to improve the national clinical practice.

**Methods:**

Nationwide online survey, involving Italian ICUs, developed by experts affiliated with SIAARTI. Invitations to participate were distributed through emails and social networks. Data were collected over a period of three months (October 1 to December 31, 2022) during 2022.

**Results:**

One hundred full responses from participating ICUs were collected. The number of beds is < 10 in most ICUs and > 20 in 11 ICUs. Most ICUs (87%) are mixed, cardiac (5%), neurosurgical (4%), or pediatric ICUs (1%). Although the nutritional program is widely prescribed based on the patients’ general evaluation, 52 ICUs (52%) do not perform nutritional risk evaluation at admission in case of > 24-h stay. Daily caloric intake is mainly based on the 25 kcal/kg equation; otherwise, the Harris-Benedict formula is mostly used, whereas indirect calorimetry is less used. Most clinicians apply a personalized nutritional approach to organ failure. Most ICUs have a nutritional management protocol, and enteral nutrition (EN) is frequently started within 2 days from admission, while supplemental parenteral nutrition is used when EN is insufficient by most clinicians. The EN administered seems to correspond to that prescribed, but it is stopped if the gastric residual gastric is > 300–500 ml in most ICUs.

**Conclusion:**

Prescription, route, and mode of administration of nutritional support seem to be in line with international recommendations, while suggestions on the tools for assessing the nutritional risk and monitoring efficacy and complications seem far less followed. Future national clinical studies are necessary to investigate the optimal nutritional and metabolic management of critically ill patients and the correspondence with the results of this survey on actual practices.

**Supplementary Information:**

The online version contains supplementary material available at 10.1186/s44158-023-00132-4.

## Introduction

In recent years, there has been much interest in the role of nutrition therapy in critical illness. Increased awareness of clinical nutrition has been hypothesized to be extremely important for intensive care unit (ICU) patients. Critical illness is associated with a catabolic stress state and an altered inflammatory response that may contribute to complications such as increased infectious morbidity, multi-organ failure, and prolonged hospitalization [1].

Careful supplementation and caloric and protein intake modulation can avoid under or overfeeding. Additionally, adequate nutritional interventions have been shown to attenuate the morbidity rate, decrease the length of hospital stay, and improve patient outcomes [2].

International guidelines have been recently updated by the American Society of Parenteral and Enteral Nutrition/Society of Critical Care Medicine [3] and the European Society of Clinical Nutrition and Metabolism (ESPEN) to integrate the best current knowledge and evidence from the literature with nutritional practices [1].

Whereas the nutritional requirements vary according to the phase of critical illness and the heterogeneity of the ICU population, these guidelines provide a set of nutrition recommendations in the most frequent clinical situations encountered in daily practice in the ICU. However, translating evidence into practice is challenging, and there is an increasing need for protocol standardization based on the latest evidence to reduce practice variation and improve the overall quality of care. A robust nutrition stewardship program could gain a reputation if the concept spreads to various national programs and regulatory guidelines released recently [4].

So far, despite these recommendations, studies have yet to assess the level of adherence to the ESPEN recommendations in the Italian context, except for a survey on nutrition support for critically ill patients during the COVID-19 pandemic [5].

This survey aimed to provide a snapshot of the current clinical practice focusing on nutritional evaluation, management, and monitoring in Italian ICUs.

In this way, the Italian ICUs might confront policies based on their clinical practice and compare these to a worldwide reference database.

## Methods

This was a nationwide online survey, developed by experts belonging to SIAARTI (the Italian Society of Anaesthesia Analgesia Resuscitation and Intensive Care) board of the Metabolism, Nutrition and Renal Therapies section, composed of five intensivists, in 2022.

The current report adheres to the Consensus-Based Checklist for Reporting of Survey Studies—CROSS reporting guideline [6] (Supplemental material 1).

### Population

In the first phase, SIAARTI distributed the questionnaire to all the Directors of Italian ICUs to be filled out by the referring physician for the nutrition and metabolism field. In the second phase, the board disseminated the questionnaire via social media to ICUs who did not answer previously with the same purpose. A short introduction and a link to the survey were available to share on social media. To avoid multiple answers from the same center, only one response was considered for each Intensive Care Unit. No monetary incentives were provided to the respondents.

Data was gathered from October 1 to December 31, 2022. No inclusion and exclusion criteria were applied for the participation to collect representative data of the national scenario. Participation was anonymous; respondents voluntarily provided clear indications about the purpose of the survey and the use of the collected data.

We used a convenience sampling strategy [7].

### Survey development

The questionnaire was based on a preliminary updated analysis of the literature on this topic and international guidelines; a panel member (AC) drafted the first version of the survey and spread it to the other members who revised and approved the final version. The board unanimously voted and approved the final formulation of the questions.

A closed structure was used to avoid multiple answers from the same respondent, and access to the questionnaire was protected by a unique, anonymous identifier assigned to each respondent. The questionnaire consisted of 30 questions in total with multiple answers.

The survey explored four domains:

1. ICU size (number of beds), ICU type (medical, surgical, cardio-surgical, neurosurgical, pediatric), hospital size, and type of hospital (academic, non-academic, private, Scientific Institute for Research, Hospitalization, and Healthcare-IRCCS) were collected in questions from 1 to 5.
2. Nutritional assessment in critically ill patients was contained in questions 6 to 13.
3. Nutritional management was investigated by questions from 14 to 21.
4. The nutritional monitoring section was collected in questions from 22 to 30.

Consistency and completion of all items were obtained using server-side techniques, such as displaying the questionnaire after submission and highlighting unanswered mandatory items The responses were reviewed and edited during a final step, displaying a questionnaire summary, and requesting confirmation until the final submission. Supplemental material 2 shows an adapted English version of the online survey.

The questionnaire was built using Survey Monkey Platinum (SurveyMonkey Inc., San Mateo, CA, USA). Respondents were instructed to answer questions from the perspective of their standard clinical practice. In case of additional questions, they could contact the board.

### Data analysis

Data was downloaded as an Excel file (Microsoft Corp, Redmond, WA, USA) and analyzed using the Jamovi software (version 1.8.4.0) for descriptive statistics. Answers were included in the analysis if Sect. 1 and at least one question from the other questionnaire sections were answered. Missing answers were included in the analysis.

The exclusion criteria included duplicated answers from the same ICU participant.

Data are presented as numbers, mean ± standard deviation (SD).

## Results

Of the 140 invited to the survey, only 100 center representatives responded. At the same time, the remaining 40 centers should have considered it appropriate, for non-specific reasons, to respond to the survey.

### Section 1: baseline characteristics

The number of beds is less than 10 in most included ICUs and over 20 in 11 Italian ICUs. Most ICUs involved (87%) are mixed, while the remaining are cardiac (5%), neurosurgical (4%), and pediatric ICUs (1%). The mean number of hospital beds considered is 458 (± 362).

### Section 2: nutritional evaluation in critically ill patients

**Fig. 1:**
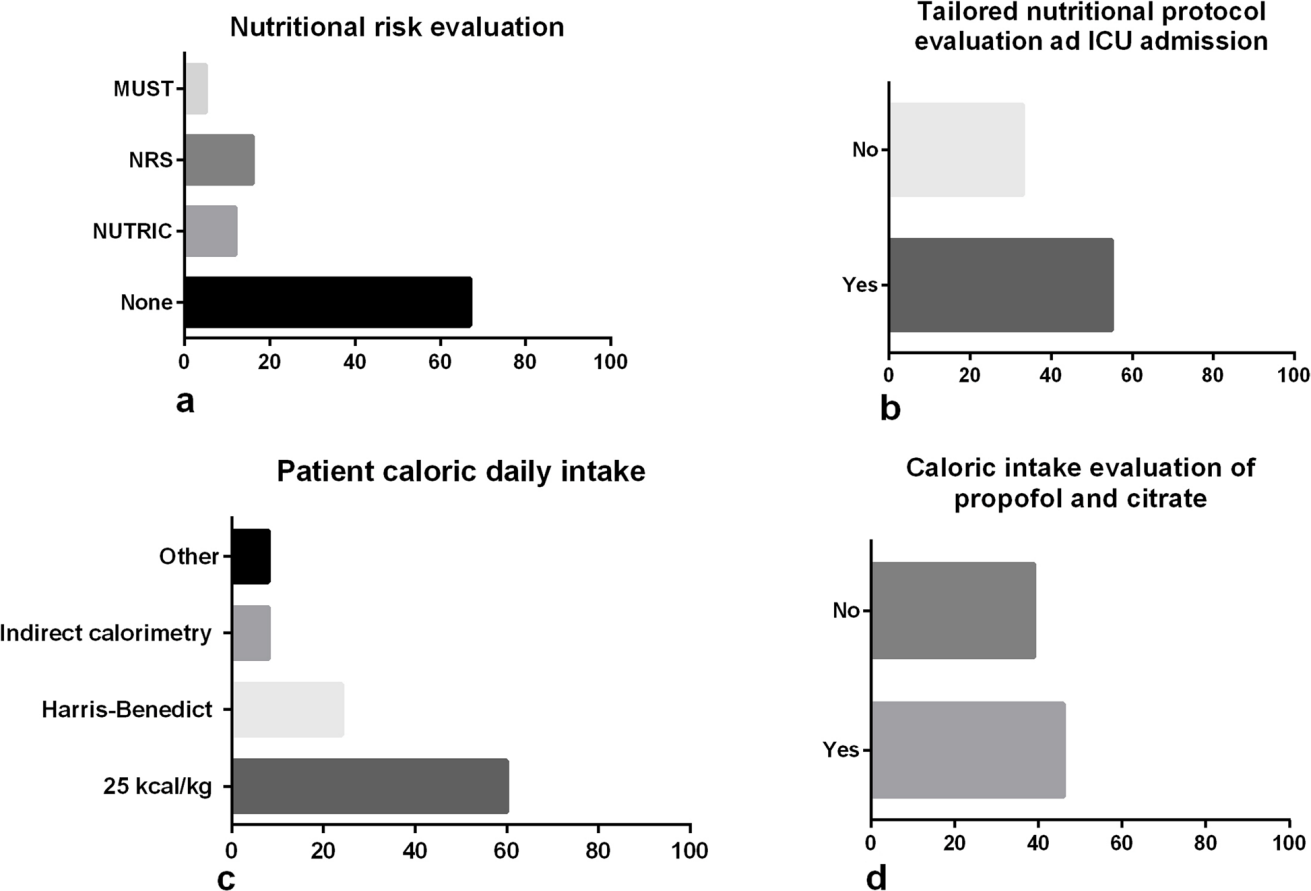
Nutritional evaluation in critically ill patients. NUTRIC nutrition risk in critically ill; *NRS*, nutritional risk Screening; *MUST* Malnutrition Universal Screening Tool

Nutritional risk evaluation at admission in case of > 24 h stay is not performed by 52% of ICUs. The nutritional program prescription based on the general evaluation of the patient (history, physical examination, and lab exams) is a widespread practice in Italy, as shown in Table 1. Interestingly, only 33% of ICUs use nutritional risk scores in their clinical practice: the NUTRIC (Nutrition Risk in Critically Ill) score is used in 12% of ICUs, and the NRS (nutritional risk Screening) is used in 16% of ICUs, while the MUST (Malnutrition Universal Screening Tool) score is used in 5% ICUs (Fig. 1A).

**Table 1:** Nutritional evaluation and monitoring in critically ill patients (Sects. 2 and 4)

Section 2. Nutritional evaluation in critically ill patients		
*Questions*	Answer	*N* = 100
Nutritional risk evaluation at the admission in case of > 24 h stay, *n* (%)	Yes	48
	No	52
Nutritional program prescription is based on (%)	Anamnesis/objective examination/lab exams	91
	Lab exams only	3
	Other	6
Time interval evaluation of nitrogen balance (NB) (%)	Never	43
	Once	42
	Twice	15
Personalized nutritional evaluation by organ failure (lungs, kidneys, liver) (%)	Yes	66
	No	34
Nutritional leader of the ICU (%)	None	22
	Intensivist	75
	Other	3
Section 4. Nutritional monitoring in critically ill		
How is glycemic control performed? (%)	Arterial blood gas analysis	48
	Capillary blood	34
	Other	6
	Missing answers	11
Glycemic target in non-diabetic critically ill patient (mg/dl) (%)	140–180 n	58
	< 140 n	24
	> 180 n	6
	Missing answers	11
Is a post-pyloric approach provided if EN is not well tolerated? (%)	Yes *n*,	46
	No *n*,	40
	Missing answers	13
EN administered equals to EN prescribed? (%)	Yes	66
	No	32
	Missing answers	11

*EN* Enteral nutrition, *ICU* Intensive care unit

Fifty-five of 100 units evaluate tailored nutritional protocol at ICU admission (Fig. 1B).

Daily caloric intake is based mainly on the 25 kcal/kg formula in 60 ICUs. The Harris-Benedict is the second formula used by clinicians in 24% of ICUs. The number of ICUs using indirect calorimetry is 8%. Another non-specified evaluation is performed in 8 ICUs.

The caloric intake from propofol and citrate is calculated by clinicians in 46% of ICUs, while it is not always calculated in 39 ICUs (Fig. 1C, D).

The following question was about the time interval evaluation of nitrogen balance, which is never used in 43% of ICUs. Among the remaining 57 ICUs, 42% measure nitrogen balance once a week per day and 15 twice.

Most clinicians perform a personalized nutritional approach to organ failure. Finally, the intensivists are the nutritional leader in 75v ICUs.

### Section 3: nutritional management in critically ill patients

**Fig. 2:**
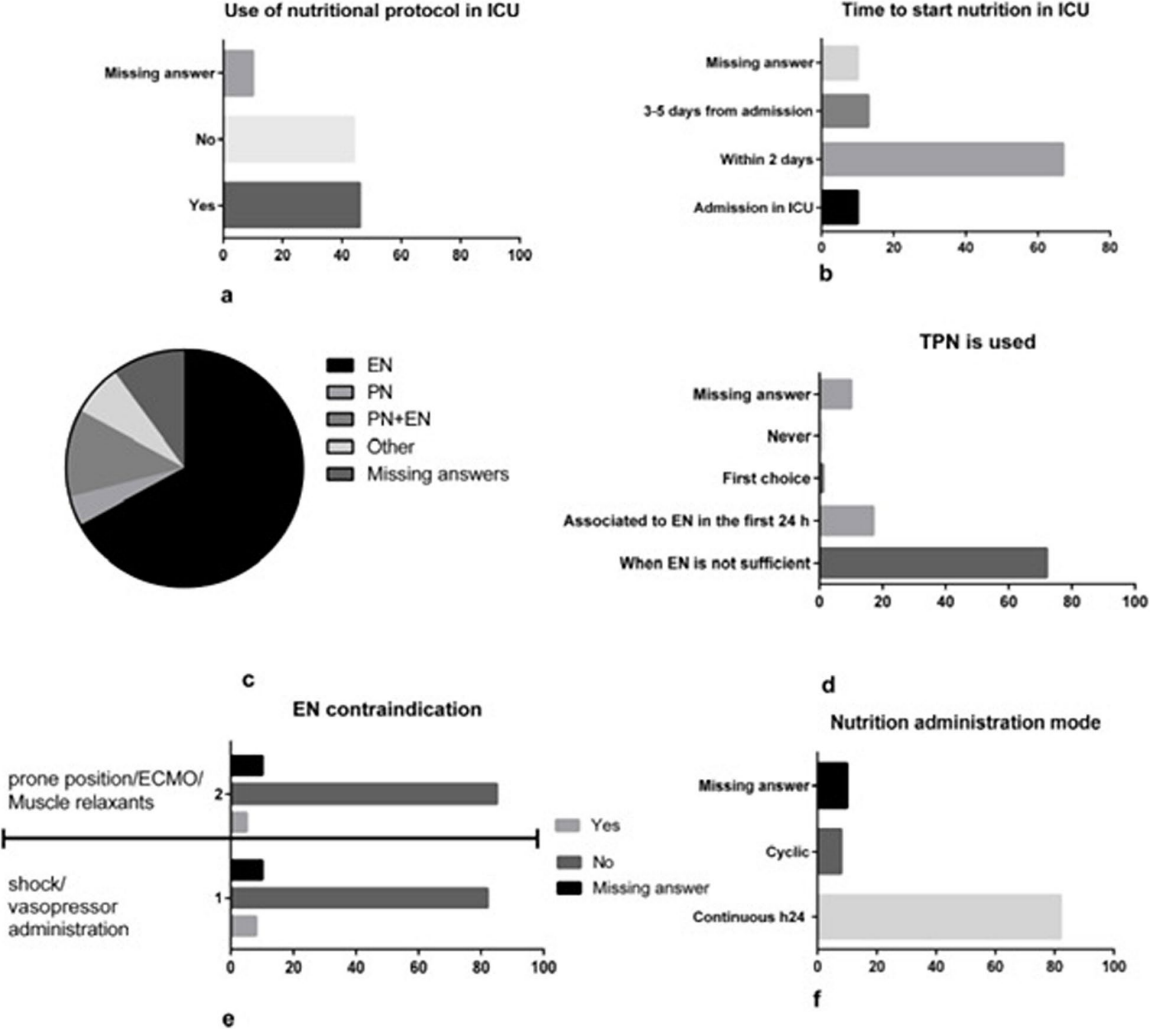
Nutrition management in critically ill patients. *TPN* total parenteral nutrition; *PN* parenteral nutrition; *EN* enteral nutrition; ECMO extracorporeal membrane oxygenation

Forty-six ICUs answered yes to using the nutritional management protocol (Fig. 2A). Enteral nutrition is frequently started within 2 days from admission in ICUs (Fig. 2B, C) Parenteral nutrition is used as a supplement when enteral nutrition is insufficient by most clinicians (Fig. 2D).

Nowadays, most clinicians do not consider any contraindications to enteral nutrition during a prone position or ECMO or muscle relaxant treatment and do not acknowledge an absolute contraindication to enteral nutrition during shock/vasopressors administration (Fig. 2E). Finally, most ICUs administer nutrition continuously (Fig. 2F).

### Section 4: nutritional monitoring in critically ill patient

**Fig. 3:**
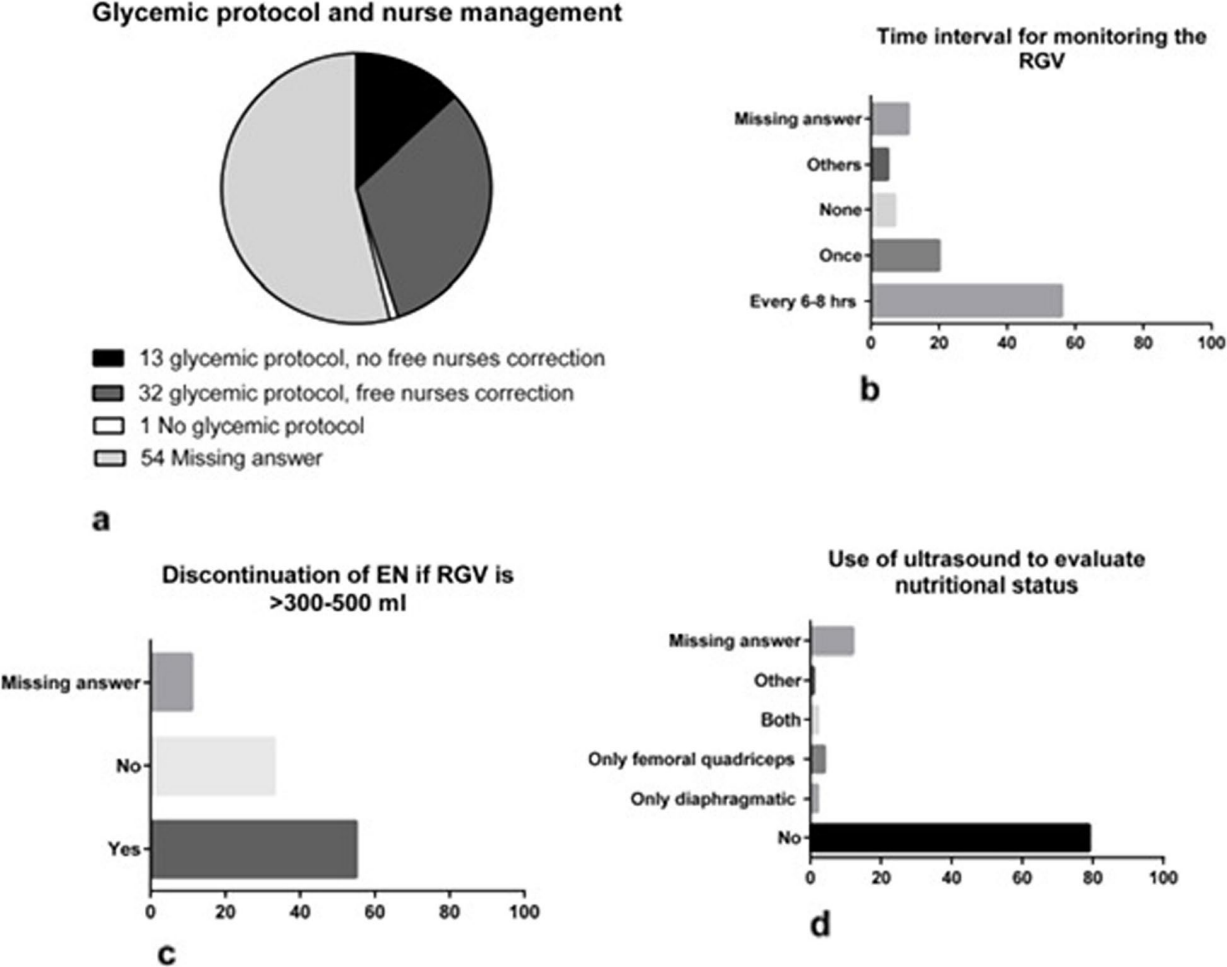
Nutritional monitoring in critically ill patients. *RGV* residual gastric volume

The last section concerns nutritional monitoring in critically ill patients (Table 1). About half of the Italian ICUs did not fill in glycaemic protocol and nurse management questions and 32% of ICUs reported using the glycemic protocol with autonomous nursing management (Fig. 3A). The glycemic level evaluation is based on arterial blood gas or capillary blood analysis in 48% or 34% of ICUs, respectively. Most clinicians refer to a glycemic target in non-diabetic critically ill patients of 140–180 mg/dl (Table 1).

Fifty-six ICUs monitor residual gastric volume every 6–8 h, while 20% of ICUs monitor it once per day (Fig. 3B).

The EN administered seems to correspond to the one prescribed, but it is stopped if the residual gastric volume is > 300–500 ml in most ICUs (Fig. 3C). When EN is not well tolerated, a post-pyloric approach is provided in 46% ICUs (Table 1).

Using ultrasound to assess a patient's nutritional status is only generally performed in some cases. If diaphragmatic ultrasound is done in only 2% of ICUs and quadriceps femoris ultrasound in 4% of ICUs, both ultrasounds are used in only 2% of ICUs. Missing answers were recorded in 12 ICUs (Fig. 3D).

## Discussion

The main findings of the current nationwide survey can be summarised as follows:

in at least half of the included ICUs, there is a standardization of clinical nutritional practice based on the use of protocols that essentially provide for the administration of EN continuously and with a non-advanced metabolic evaluation of the patient. Furthermore, glycemic control is entirely the prerogative of the nursing staff in less than half of the ICUs.

Determination of nutritional status is not a straightforward process, and the recently developed Global Leadership Initiative on Malnutrition (GLIM) criteria consider the coexistence of phenotypic and etiologic criteria [8]. However, in critically ill patients, the diagnosis of malnutrition is made difficult by the challenges of determining food intake and weight loss. Indeed, since nutritional support aims to preserve muscle mass in patients without malnutrition, the nutritional risk is even more important than nutritional status, which is often determined by the severity of the disease with no regard to nutritional status [8].

Even though a general clinical assessment has been recommended to assess malnutrition in ICU patients [2], most ICUs do not evaluate the nutritional risk at the ICU admission. A lack of awareness of the importance of early recognition and treatment of malnutrition among healthcare team members remains a significant challenge, particularly in the intensive care setting.

So far, several tools have been developed for nutrition screening and assessment of hospitalized patients and the modified Nutrition Risk in the Critically Ill (mNUTRIC) has been suggested for the nutritional risk assessment of critically ill patients [2, 9, 10].

We found that most ICUs do not use any score. Among all screening tools, NRS and NUTRIC scores are mainly used, maybe because they are the easiest and quickest to calculate and have the most robust predictive value for mortality [11, 12].

However, the indication to tailoring the nutritional therapy to minimize under or overfeeding is widespread in all Italian ICUs. Based on the results of this survey, predictive equations remain the most common resting energy expenditure (REE) estimation method. Commonly used in clinical practice is 25 kcal/kg.

Importantly, predictive equations tend to over or under-estimate REE with an accuracy rate, defined as % of patients where the predicted value by the equation is within 10% of the measured value by indirect calorimeters (IC), of 12% for 25 kcal/kg and 30% for Harris-Benedict in critically ill setting [13].

IC is still unavailable in most Italian ICUs, whereas it is the gold standard for determining REE [14]. Factors limiting the reliability and feasibility of IC measurements are agitation, fever, sedatives, and vasoactive adjustments. Likewise, air leakages in respiratory circuits, mechanical ventilation with PEEP > 10 or with FiO2 > 80%, non-invasive ventilation, ECMO, dialysis, or continuous renal replacement therapy [14].

The accuracy of caloric intake evaluation can further decrease if propofol, citrate, and dextrose intake are not considered [2, 15, 16].

The evaluation of nitrogen balance (NB) is generally performed once a week in ICUs. The NB could be considered an excellent marker to establish dietary protein requirements in critically ill patients whereas it did not appear to predict clinical outcomes [17, 18]. The latest recent meta-analysis showed that improved NB was associated with all-cause mortality in critically ill patients [19]. This highlights the requirement for dynamic monitoring of NB during nutrition treatment [19].

The nutritional evaluation is personalized according to the potential organ failure of critically ill patients in most ICUs and the intensivist is the nutrition leader. According to a recent study, critical care physicians’ knowledge and understanding of nutritional therapy are limited, especially in supportive preparation [20].

In the future, the continuing education of all intensivists, rather than only the leader, should emphasize the comprehensiveness and importance of nutritional management and encourage them to cooperate with dietitians to promote the development of protocols and standardization of therapy.

The use of the ICU nutritional protocol in Italy could be debated. Recommendations for medical nutritional therapy in critically ill patients vary among guidelines [1–3, 21]. For these reasons, implementing specific recommendations into clinical routine remains often insufficient.

The scientific community still debates when to start nutrition in critically ill patients. According to guidelines, nutrition treatment usually begins within 2 days from admission [1].

EN is the nutrition of choice in the first days from admission in the ICU for more than half of ICUs. Supplemental parenteral nutrition (SPN) is used in most cases when EN is insufficient. At the same time, in a small percentage of cases, PN is associated with EN in the first 24 h, and rarely, TPN is the first nutritional choice for ICU patients.

Regarding some particular issues in critical illness, the guidelines favor early EN in patients receiving ECMO, prone positioning, and muscle relaxants because it reduce infectious complications. Our survey showed that this indication seems fully respected and well-known [1] and physician respondents know the indication of nutrition routes in shock conditions and hemodynamic alterations [1].

Furthermore, the preferred way of nutrition administration is continuous infusion, as suggested by the guidelines [1]. Another important item investigated by the survey is patients’ glycaemic status and its management by nurses. From the obtained answers, most ICUs use a glycaemic protocol according to which nurses correct glycemia on their own. The most used glycaemic target in non-diabetic critically ill patients is 140–180 mg/dl.) [1].

Besides, blood glucose sample is drawn mainly from arterial blood gas analysis whereas the ESPEN Guidelines suggest how blood should preferentially be drawn from central venous or arterial blood, avoiding capillary pricks in critically ill patients as several sources of interference are likely [2, 22, 23].

Other topics analyzed by the survey are the management of residual gastric volume and EN feeding intolerance. In several studies, the frequency of RGV measurement was every 6–8 h [16, 17]. However, the need to be more consensus about the rate of RGV threshold persists [24, 25]. In almost all ICUs, the dose of nutrition prescribed is the actual nutrition administered. In the case of RGV > 300–500 ml, the most common behavior in ICUs is to stop EN. ESPEN states enteral feeding should be delayed when RGV is > 500 mL/6 h. Furthermore, in patients with gastric feeding intolerance not solved with prokinetic agents, post-pyloric feeding (mainly jejunal) should be used, especially in patients at high risk for aspiration [1]. In Italy, the post-pyloric approach is common in ICUs.

In our study, ICUs still lack confidence regarding the use of ultrasonography (US) to assess a patient’s nutritional state. Indeed, diagnosing both malnutrition and sarcopenia requires assessing lean body mass with validated methods. Critically ill patients can lose up to 15% of their total muscle mass in the first week of stay [26], which has been associated with detrimental long-term effects. The monitoring of lean body mass with validated methods has then been suggested as a critical component of the assessment of critically ill patients, to assess the current muscle mass for the nutritional diagnosis and risk stratification, to monitor the progression of muscle loss and/or recovery of muscle mass and to evaluate the success or failure of therapeutic interventions [27].

In a recent study, US muscle mass assessment was able to detect short-term changes in critically ill patients and it was also identified as a useful follow-up tool [28–30].

Body composition assessment is a relatively new practice in the intensive care field. Despite some technical limitations in critically ill patients, their use is steadily increasing, and the survey findings pave the road for planning educational interventions to spread further the application of these tools besides the research field.

## Strengths and limitations

This online survey enabled the collection of anonymized information and facilitated the data collection from Italy. The survey was disseminated through the email list of ICU medical directors available to SIAARTI and social media to reach all Italian ICUs. However, our survey might not precisely reflect all ICUs because several units still need to answer the call or complete the questionnaire. Another limitation is that the advertisement through social media could have caused selection bias as the physician who does not use social media could not have taken the survey.

Furthermore, especially after the COVID-19 pandemic, an updated census of Italian ICUs seems desirable, as the list available to SIAARTI is likely outdated because of the organizational changes that occurred due to the pandemic.

## Conclusion

In conclusion, the prescription, route, and mode of administration of nutritional support comply with international recommendations. In contrast, the suggestions on the tools for assessing the nutritional risk and monitoring the efficacy and the complications seem far less followed. Future national clinical studies would be useful to investigate the clinical approach to critically ill patients in terms of nutrition and metabolic management, as well as the correspondence between what is reported in the survey and actual practices. The survey of nutrition and metabolism can further evolve with the contribution of the Italian Society of Anesthesia Analgesia Resuscitation and Intensive Care (SIAARTI) and other Italian Societies of Nutrition to fill in current existing gaps in knowledge and to support decreasing diversity in nutrition care practices through collaborations and new evidence.

### Supplementary Information

**Additional file 1.** Supplementary material 1. CROSS Checklist.

**Additional file 2.** Supplementary material 2. Questionnaire.

## Data Availability

The database used and analyzed during the current study is available from the corresponding author on reasonable request.

## Abbreviations

ICU: Intensive care unit
EN: Enteral nutrition
TPN: Total parenteral nutrition
PN: Parenteral nutrition
ECMO: Extracorporeal membrane oxygenation
RGV: Residual gastric volume
NUTRIC: Nutrition risk in critically ill
NRS: Nutritional risk screening
MUST: Malnutrition Universal Screening Tool
