# Supplementary material for: Nutritional support and prevention of post-intensive care syndrome: the Italian SIAARTI survey
Source: J Anesth Analg Crit Care. 2023 Nov 7;3:45. doi: 10.1186/s44158-023-00132-4 (PMC10631207; doi:10.1186/s44158-023-00132-4)
Supplement: Supplementary file 2 — Additional file 2. Supplementary material 2. Questionnaire. [file 44158_2023_132_MOESM2_ESM.pdf]

Section 1: Characteristics of the Centre

1. Region:
2. Structure:
    a. Hospital;
    b. University Hospital;
    c. Private clinic;
    d. IRCCS.

3. Number of beds in Intensive Care:
4. Total number of beds in the hospital:

5. Type of Resuscitation:
    a. General;
    b. Cardiac surgery;
    c. Neurosurgical;
    d. Pediatric;
    e. Other.

Section 2: Nutritional Evaluation to the Critically Ill Patient

6. Is a nutritional risk evaluation performed at the admission in ICU in case of >24 hours?
    a. Yes;
    b. No.

7. The prescription of a nutritional program is based on:
    a. Anamnesis, physical examination of the patient and laboratory tests;
    b. I prefer to evaluate only laboratory tests.
    c. Other

8. What score is used for nutritional risk assessment?

   a. Nutritional Risk Score, NRS;

   b. NUTRIC score;

   c. MUST score;

   d. No score.

9. Is the nutrition assessment performed at patient admission in ICU and, if necessary, drawn up a nutritional program?

   a. Yes;

   b. No.

10. How are the patient's caloric needs assessed?

    a. Indirect calorimetry;

    b. Harris-Benedict formula;

    c. Faisy equation;

    d. 25 Kcal/kg;

    e. Penn State equation.

11. Does the caloric intake evaluation take into account a possible contribution of propofol, glucose, citrate, and lactate?

    a. Yes;

    b. Not always;

    c. I don't have time to make such an accurate assessment;

    d. Never.

12. How often is the evaluation of the nitrogen balance carried out?

    a. Once a week;

    b. Twice a week;

    c. It is not carried out.

13. Is a personalized nutritional evaluation performed by organ failures (lungs, kidney, liver)?

a. Yes;

b. No.

14. Who is the nutritional leader in ICU?

a. An intensive care Physician;

b. A nutritionist;

c. Other professional figure;

d. None.

Section 3: Nutritional Management to the Critical Patient

15. Is a nutritional protocol used in your intensive care unit?

a. Yes;

b. No.

16. When is the administration of nutritional therapy started in your intensive care unit?

a. Within the first 2 days of admission;

b. Within the first 5 days of admission;

c. After the first week after hospitalization;

d. Upon admission to intensive care.

17. What type of nutrition is chosen in the first days of ICU?

a. Enteral nutrition (EN);

b. Total parenteral nutrition (TPN);

c. EN + SPN;

d. None, only electrolyte solution associated with carbohydrates.

18. When is TPN provided in your intensive care unit?

a. As a first-choice (TPN only);

b. As supplemental nutrition in the first 24-48 hours after admission (EN+SPN);

c. As supplemental nutrition when the caloric-protein intake provided with EN alone is inadequate (intolerance or contraindications);

d. Never.

19. Do you think that the prone position, ECMO treatment, muscle relaxant administration represents a contraindication to EN?

a. Yes;

b. No.

20. Do you think that shock, vasopressors is an absolute contraindication to EN?

a. Yes;

b. It is not an absolute contraindication because it depends on the hemodynamic stability of the patient.

21. How is enteral nutritional therapy administered?

a. Repeated boluses;

b. Cyclic (a few hours a day);

c. Continue within 24 hours.

Section 4: Nutritional Monitoring to the Critically Ill

22. Is there a glycemic control protocol in your intensive care unit?

a. Yes;

b. No;

23. If so, is glycemic management performed independently by the nursing staff?

a. Yes;

b. No.

24. How is glycemic control performed?

a. Blood gas analysis;

b. Peripheral venous blood sample;

c. Central venous blood sample;

d. Capillary blood;

25. What is the glycemic target in the critical non-diabetic patient in your intensive care?

a. <140 mg/dl;

b. 140-180 mg/dl;

c. >180 mg/dl;

26. What is the frequency of residual gastric monitoring in your intensive care?

a. Once/day;

b. Every 6-8 hours;

c. None;

d. others.

27. In case of residual gastric volume >300-500 ml, EN is stopped?

a. Yes;

b. No.

28. Is a post pyloric approach provided if EN is not well tolerated?

a. Yes;

b. No.

29. Is enteral nutrition administered equals to EN prescribed?

a. Yes;

b. No.

30. In your intensive care, is ultrasonography used to assess the nutritional state?

a. Yes, diaphragm only

b. Yes, only quadriceps femoris muscle

c. Both ultrasonography of the quadriceps femoris muscle and diaphragm

d. No.
